# Thymax, a gross thymic extract, exerts cell cycle arrest and apoptosis in Ehrlich ascites carcinoma in vivo^☆^

**DOI:** 10.1016/j.heliyon.2022.e09047

**Published:** 2022-03-05

**Authors:** Nariman K. Badr El-Din, Azza I. Othman, Maggie E. Amer, Mamdooh Ghoneum

**Affiliations:** a Department of Zoology, Faculty of Science, University of Mansoura, Mansoura 33516, Egypt; b Department of Surgery, Charles R. Drew University of Medicine and Science, Los Angeles, CA, USA; c Department of Surgery, University of California Los Angeles, Los Angeles, CA, USA

**Keywords:** Thymax, Cell cycle, Apoptosis, Ehrlich carcinoma, in vivo

## Abstract

Thymax is a gross thymic extract that has been shown to induce apoptosis in vitro for human breast cancer cells. Here we examine Thymax's ability to induce apoptosis in animals bearing Ehrlich ascites carcinoma (EAC). Thymax was administered six days/week orally to mice (5.45 mg/kg body weight) beginning either 14 days prior to EAC inoculation or 9 days post inoculation; treatment continued for 30 days post inoculation. Pretreatment of mice with Thymax markedly delayed tumor growth and reduced tumor incidence by 38.9%, and tumor volumes relative to untreated controls were suppressed by 90.5% and 55.0% for pre- and post-inoculation groups, respectively. Treatment with Thymax inhibited cellular proliferation by decreasing the expression of tumor markers Ki-67, PCNA, and Cyclin D1 in cancer cells and increasing the expression of p21 and p27. This was associated with the ability of Thymax to arrest the cell cycle of EAC cells in the G0/G1 phase and to induce apoptosis, as indicated by a significant increase in the sub-G1 phase's percentage of hypodiploid cells and further affirmed by DNA fragmentation and Annexin V/propidium iodide staining. In addition, Thymax exerted its apoptotic effect in EAC cancer cells through a mitochondrial-dependent pathway, as evidenced by an increased Bax/Bcl-2 ratio, up-regulation of p53 expression, and activation of caspase-3. We conclude that Thymax supplementation enhances tumor cell demise by arresting the cell cycle and inducing apoptosis. These data suggest that Thymax could be a new adjuvant for breast cancer treatment.

## 1 Introduction

Cancer is the second largest cause of mortality worldwide, resulting in over 9.5 million deaths in 2018 [1,2]. Cancer incidents remain high across all income levels, with low- and middle-income countries exhibiting the highest mortality rates [3]. It is estimated that annual worldwide cancer diagnoses will rise to 27.5 million new cases by the year 2040 [2]. Conventional cancer treatments like chemotherapy are associated with dangerous toxicities and lowered quality of life for patients. There is an urgent need to find alternative treatments that can improve patients’ health with fewer side effects. One of the most promising treatments that has already been used to improve health for more than a century is the thymus.

The thymus is a vitally important organ that carries out several biological effects such as immune reactivity and immunologically competent T-cell production. The thymus is the site in which progenitor cells are created, mature, and differentiate into mature T cells. These mature T cells then emigrate and comprise the peripheral T cells that coordinate numerous aspects of the adaptive immune system [4]. However, the thymus undergoes age involution. Recent work has shown that aged thymic involution leads to architectural changes of the thymus and a decrease in tissue mass in almost all vertebrates from birds to teleosts, amphibians, reptiles, and humans, indicating that this process is evolutionarily ancient and conserved [5, 6, 7].

Numerous scientists have attributed the inability of the immune system to recover after serious insults to thymic involution. For the elderly, immunosuppressive interventions post treatment with chemotherapy and ionizing radiation and exposure to infections (e.g. hepatitis C, HIV) result in increased mortality and morbidity. Therefore, research has been directed toward developing effective and safe strategies for thymus rejuvenation in clinical settings [8] or transplanting the thymus [9, 10]. Transplanting thymic tissue or using thymic hormones can partially restore the diminished proliferation of T lymphocytes or thymic involution [11, 12].

The ability of thymus to regulate immune cell transformation and selection is related to thymus production of several self-hormones such as thymus humoral factor, thymopentin, thymulin, and thymosin. Thymosin α1 (Tα1) has been shown to act as a chemopreventive agent in animal models by preventing mammary carcinogenesis, reducing the number of lung adenomas and increasing animal survival [13]. Furthermore, patients with different malignancies have been treated with Tα1, including for lung [14] and renal cancer [15] and for melanoma [16, 17]. Thymosin fraction 5 (TF5) inhibits growth of hematopoietic leukemia cell lines and neuroendocrine tumor cells [18, 19, 20], and thymic extracts have been found to exert immunomodulatory effects, including stimulation and/or maturation of T-cell differentiation [21] as well as the activation of dendritic cells (DC) [22] and natural killer (NK) cells [23].

Although thymic extracts are heavily investigated, the molecular mechanisms underlying their anticancer activity remain unclear. Here we introduce another thymic product called Thymax, a gross thymic extract [24]. Our earlier studies have shown that Thymax can correct the functional decline of murine immune cells associated with age [25] and activate human monocyte-derived DCs [26]. In addition, we have demonstrated that Thymax exerts an apoptotic effect in vitro in human breast cancer cells (MCF-7) [24], where apoptosis induction was linked with the activation of caspases 8 and 9 and with a decrease in mitochondrial polarization. In the current study, we test the hypothesis that Thymax exerts anticancer effects in vivo, in part via inhibition of cell cycle progression and in part via activation of the mitochondrial pathway of apoptosis.

## 2 Materials and methods

### 2.1 Thymax

Thymax is a gross thymic extract composed of thymosin, thymomodulin, and many other peptides. It is characterized by heat-resistant peptides contained in acidic pH (via treatment with L-ascorbic acid and NaCl) and unique from other thymus extracts in its composition and its ability to be orally administered into the body. Work is underway to identify Thymax's active factors. Thymax was kindly manufactured and provided by YS Nature Company, Tokyo, Japan. More details about its preparation may be found in [24].

### 2.2 Preparation of Ehrlich ascites carcinoma (EAC) cells and induction of tumor development

EAC was selected because it originates from the mouse and is easily transplantable into immunocompetent mice. Breast cancer has been studied over the last four decades through the well-established mouse model of EAC. EAC is originally hyperdiploid; it is an undifferentiated carcinoma with unique characteristics, including rapid proliferation, high transplantable capability, short life span, and 100% malignancy [27, 28, 29, 30]. EAC cells were procured from the National Cancer Institute, Cairo University, Egypt. We followed previously established models to study EAC in mice [28, 29, 30, 31, 32, 33, 34]. Briefly, viable EAC cells of a fixed number from donor mice were diluted in saline (0.2 ml of EAC, consisting of 2.5 × 10^6^ viable cells/mouse) and injected intramuscularly into animals’ right thighs for solid tumor development.

### 2.3 Animals

54 female Swiss albino mice (19–21 g weight, 2 months old) were purchased from the National Cancer Institute, Cairo University, Egypt. They were acclimatized for one week at our animal research facility, then kept in pathogen free cages in alternating 12-h light and dark cycles with constant temperature (24 ± 2 °C) and 10% relative humidity. Standard cube pellets (Misr Oil & Soap Company, Cairo, Egypt) and water were provided to mice ad libitum. Animal protocols complied with the University of Mansoura, Egypt's Guide for the Care and Use of Laboratory Animals. The study was approved by the University of Mansoura, Egypt's Committee on the Ethics of Animal Experiments, approval number Sci-Z-Ph-2020-22*.*

### 2.4 Experimental design

Mice were orally administered Thymax (5.45 mg/kg body weight) 6 times/week throughout the study. We conducted a prior preliminary study on mice administered different Thymax doses and found that 5.45 mg/kg body weight was an optimal Thymax dose (Supplementary File 1). Mice were separated randomly into three groups with 18 mice/group: Group 1 (Inocul Control): tumor-bearing mice with no Thymax treatment; Group 2 (Thymax Pre-Inocul): mice administered Thymax 14 days before tumor inoculation, with Thymax treatment continuing to Day 30 post tumor inoculation; and Group 3 (Thymax Post-Inocul): mice administered Thymax 9 days after tumor inoculation, with Thymax treatment continuing to Day 30 post tumor inoculation. Parameters under investigation included tumor growth, PCNA and Ki-67 expression in tumor cells, apoptosis, cell cycle progression, apoptotic and cell cycle regulators’ expression, and DNA damage.

### 2.5 Tumor incidence and growth

Thymax's antitumor activity was assessed daily by checking for palpable tumors and measuring tumor volume changes (TV/mm³).

#### 2.5.1 Tumor volume (TV)

TV was measured by digital Vernier calipers 3 days/week, beginning on Day 9 after EAC inoculation and continuing to Day 30. The following formula was used to calculate TV: TV (*mm*^3^) = 0.52AB^2^, where A is the minor axis and B is the major axis.

### 2.6 Sample collection

On Day 30 (experiment end), mice were anesthetized with sodium pentobarbital (40 mg/kg BW, i.p.) and sacrificed by cervical dislocation. Solid tumors were extracted by dissection and tumor tissues were either frozen immediately until performing different investigations or embedded in paraffin for immunostaining processing.

### 2.7 Flow cytometric analysis

#### 2.7.1 Cell preparation

Tumor tissues were extracted from animals, divided into small pieces, and rubbed through nylon gauze (40–50 mesh count/cm, HD 140 Zuricher Buteltuch fabrik AG), following which samples were washed with Tris–ethylenediaminetetraacetic acid (Tris-EDTA) buffer at pH 7.5 [3.029 g of 0.1 M Tris-(hydroxymethyl aminomethane), 1.022 g of 0.07 M HCl, 0.47 g of 0.005 M Tris-EDTA] through the gauze. Phosphate buffered saline (PBS) was used to suspend cells, and this was centrifuged at 4 °C for 5 min at 200–300 g, resuspended in sterile PBS (1 × 10^6^ cells/ml), and fixed and permeabilized with ice-cold 70% ethanol in PBS, then kept in the same fixative at -20 °C until analyzed.

#### 2.7.2 Cell cycle analysis by propidium iodide

Tumor cell suspensions (1 × 10^6^ cells/ml) were centrifuged (300 x g) for 5 min before cell pellets were resuspended in 1 ml propidium iodide (PI) solution (Sigma-Aldrich): (25 μg/mL PI [wt/vol], 0.1 Mm ethylenediaminetetraacetic acid, and 10 μg/mL RNase A [wt/vol] in PBS) for 30 min in the dark. Flow cytometry (Accuri C6; BD Biosciences) was then performed using Accuri C6 software for analysis. This software was used to calculate the G0/G1 peak's coefficient of variation and the cell percentages in each cell cycle phase of the DNA (G0/G1, S, and G2/M). This also allowed for calculating the ratio of the apoptosis index (AI) to the proliferation index (PrI).

#### 2.7.3 Detection of apoptosis by AnnexinV/PI double staining

Early and late apoptosis induced by Thymax in tumor cells (cell density = 1 × 10^6^ cells/ml) were identified and quantified by flow cytometry using fluorescein isothiocyanate conjugated Annexin V/propidium iodide (PI) apoptosis detection kit (BD Biosciences, USA). Cell suspension in binding buffer was mixed with 5 μl of Annexin V-FITC and 5 μl of PI and incubated for 15 min at room temperature in the dark according to the manufacturer's instructions, following which it was analyzed within 1 h using Accuri C6 software (BD Biosciences). Early apoptotic cells, late apoptotic cells, and necrotic cells were ascertained by fluorescence color and location of presentation in fluorescence-activated cell sorting histograms.

#### 2.7.4 Expression of cell cycle progression, apoptosis, and cell proliferation related proteins

Antibodies against p53 (cat. no. sc-98), p21 (cat. no. sc-6246), p27 (cat. no. sc-1641), Bax (cat. no. sc-7480), Bcl-2 (cat. no. sc-7382), caspase-3 (cat. no. sc-7272), cyclin D1 (cat. no. sc-20044), PCNA (cat. no. sc-56), Ki-67 (cat. no. sc-7846) protein, and other reagents were purchased from Santa Cruz Biotechnology, Inc., Dallas, TX, USA. Tumor cells (1 × 10^6^) from Thymax-treated mice were incubated with the appropriate antibody (1 μg per 1 × 10^6^ cells) for 1 h at room temperature, followed by 20 min incubation at room temperature with the respective FITC-secondary antibody. Cells were then thoroughly washed with PBS with 1% bovine serum albumin (BSA) and analyzed on a flow cytometer (Accuri C6; BD Biosciences, CA, USA). 20,000 total cells were analyzed via Accuri C6 software (BD Biosciences) to plot FITC-fluorescence vs. counts in logarithmic fluorescence intensity.

#### 2.7.5 Western blotting analysis

Tumor tissues were lysed in RIPA buffer (cat. no. sc-24948; Santa Cruz Biotechnology, Inc.) containing protease inhibitors, and the Bradford method was used to determine protein concentrations [35]. Proteins were loaded in equal amounts (20 μg) and separated on 5% SDS-PAGE gel. They were then transferred to a 0.45-μm polyvinylidene fluoride (PVDF) membrane (EMD Millipore). After blocking nonspecific binding sites for 1 h with 5% nonfat milk at room temperature, samples were incubated at 4 °C overnight with the respective primary antibodies and with β-actin as the loading control (the dilution was 1:1000 5% BSA in TBST). The primary antibodies used were antibodies against p53 (cat. no. sc-98; 1:100), Bcl-2 (cat. no. sc-7382; 1:200), Bax (cat. no. sc-7480; 1:200), caspase-3 (cat. no. sc-7272; 1:200), p21 (cat. no. sc-6246; 1:200), p27 (cat. no. sc-1641; 1:200), cyclin D1 (cat. no. sc-20044; 1:200), all from Santa Cruz Biotechnology, Inc., and β-actin (cat. no. NBP1-47423; Novus Biologicals, Ltd.). The molecular weights for p53, p21, p27, cyclin D1, Bax, Bcl-2, caspase-3, and β-actin were 53 kDa, 21 kDa, 28 kDa, 37 kDa, 23 kDa, 26 kDa, 0.34 kDa, and 0.43 kDa, respectively. After washing three times in Tris-buffered saline and 0.1% Tween-20 for 10 min each time, membranes were incubated with horseradish peroxidase (HRP)-conjugated bovine mouse IgGκ binding protein (m-IgGκ-BP-HRP: cat. no. sc-516102; Santa Cruz Biotechnology, Inc.) which was diluted at 1:5000 for 1 h at room temperature. Tetramethylbenzidine (TMB; Sigma-Aldrich: Merck KGaA) was used to develop the specific protein bands. The densitometric analysis of protein bands was carried out using ImageJ software 1.42q on a 64-bit Windows 7 operating system. β-actin was used to normalize the density of each band.

#### 2.7.6 Immunohistochemical detection of PCNA and Ki-67 expressions

Tumor tissues were collected and processed for immunostaining evaluations. Briefly, 4-μm-thick, paraffin-embedded tumor tissue sections were cut at room temperature onto positive-charged slides, deparaffinized, hydrated through series of alcohols, microwaved in 10 Mm citrate buffer (pH 6.0) for antigen retrieval and treated with hydrogen peroxide to quench endogenous peroxidases. After blocking non-specific binding, sections were incubated overnight at 4 °C with anti-PCNA (cat. no. sc-56; 1:200) or anti-Ki-67 (cat. no. ab15580; 1:500) antibody obtained from Santa Cruz Biotechnology, Inc., and Abcam, respectively. Subsequently, sections were treated with secondary antibody for 2 h at room temperature, biotinylated rabbit anti-mouse antibody IgG (cat. no. ab7074, 1:200, Abcam) for PCNA or biotinylated goat anti-rabbit IgG (cat. no. ab6720, 1:200, Abcam) for Ki-67 followed by conjugated horseradish peroxidase streptavidin (cat. no. ab64269, Abcam) for 10 min at room temperature. Finally, slides were placed in 3,3′ diaminobenzidine (DAB) and incubated for 10 min at room temperature to visualize reactions until desired color was achieved. Mayer's Hematoxylin was applied as a counterstain to the sections for 1 min at room temperature, and the slides were then examined by light microscopy at x200 and x400 magnification. Protein expression was detected in the nuclei of tumor tissues. Positive signals were indicated by a brown color. Assessment of PCNA and Ki-67 labeling indices was dependent upon the positive cell proportion and the staining intensity. For each specimen, five areas were randomly chosen and the average number of positive cells in each area were counted with Image J Software 1.42q on a Windows 7 64-bit install [36].

#### 2.7.7 Detection of DNA damage by gel electrophoresis

A TIANamp Genomic DNA Kit (Tiangen Biotech (Beijing) Co., Ltd) was used to extract DNA from tumor tissues according to the manufacturer's instructions. DNA sample purification and visualization was performed with standard gel electrophoresis in a 1% agarose gel, separating DNA by size (for example, by base pair length). DNA segment lengths were determined using a VC 100bp Plus DNA Ladder (cat. no. NL1405, Vivantis Technologies Sdn. Bhd.). Gels were analyzed with Gel analyzer Pro v3.1 (gel-pro-analyzer.software.informer v3.1) to automatically detect lanes and bands, and were subsequently further analyzed quantitatively with ImageJ v1.48 software.

#### 2.7.8 Statistical analysis

Reported values are stated as mean ± SE. To determine the significance among multiple groups, one-way analysis of variance (ANOVA) with post hoc test (LSD) was used. For the tumor volume measurements, a mixed two-way ANOVA was used, with treatment day as within-subjects factor and treatment type as a between-subjects factor; differences between specific groups at each time point were evaluated by post hoc Bonferroni main effect analysis. P-values less than 0.05 were considered statistically significant.

## 3 Results

### 3.1 Effect of Thymax on tumor incidence and tumor volume (TV)

The effect of Thymax supplementation on tumor incidence and TV in mice over 30 days was monitored and examined.

#### 3.1.1 Tumor incidence

All mice that were inoculated with EAC cells alone (Inocul Control) developed tumors by day 9. Meanwhile, Thymax Pre-Inocul mice recorded tumor incidence in only 16.6% of animals (3/18). Tumors developed slowly in this group and tumor incidence was reduced by approximately 39% of the animals (11/18) on day 30 (data not shown).

#### 3.1.2 Tumor volume (TV)

**Figure 1:**
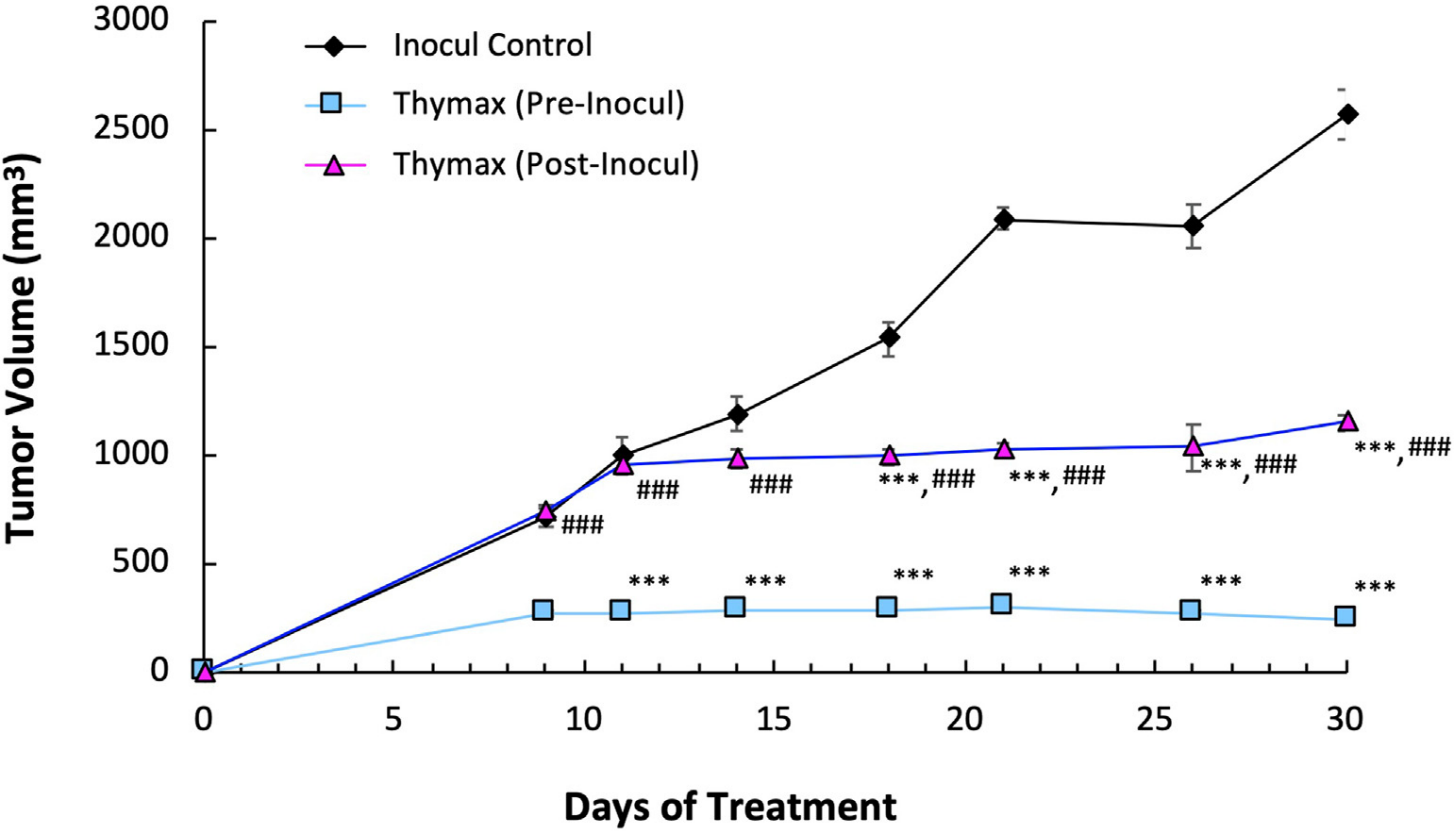
Effect of Thymax on tumor volume (mm^3^) was recorded at different intervals for 30 days. Data at different time points are expressed as mean ± SE. ∗∗∗ Significantly different from the Inocul Control group at *p < 0.001* level. ^###^ Significantly different from the Thymax (Pre-Inocul) group at *p < 0.001* level.

Figure 1 shows that treatment with Thymax pre-inoculation (Thymax Pre-Inocul) resulted in a profound decrease by 90.5% in TV 30 days after tumor cell inoculation relative to the Inocul Control group, while Thymax post-inoculation (Thymax Post-Inocul) showed a decrease of 55% of control.

### 3.2 Cell cycle progression and apoptosis

**Figure 2:**
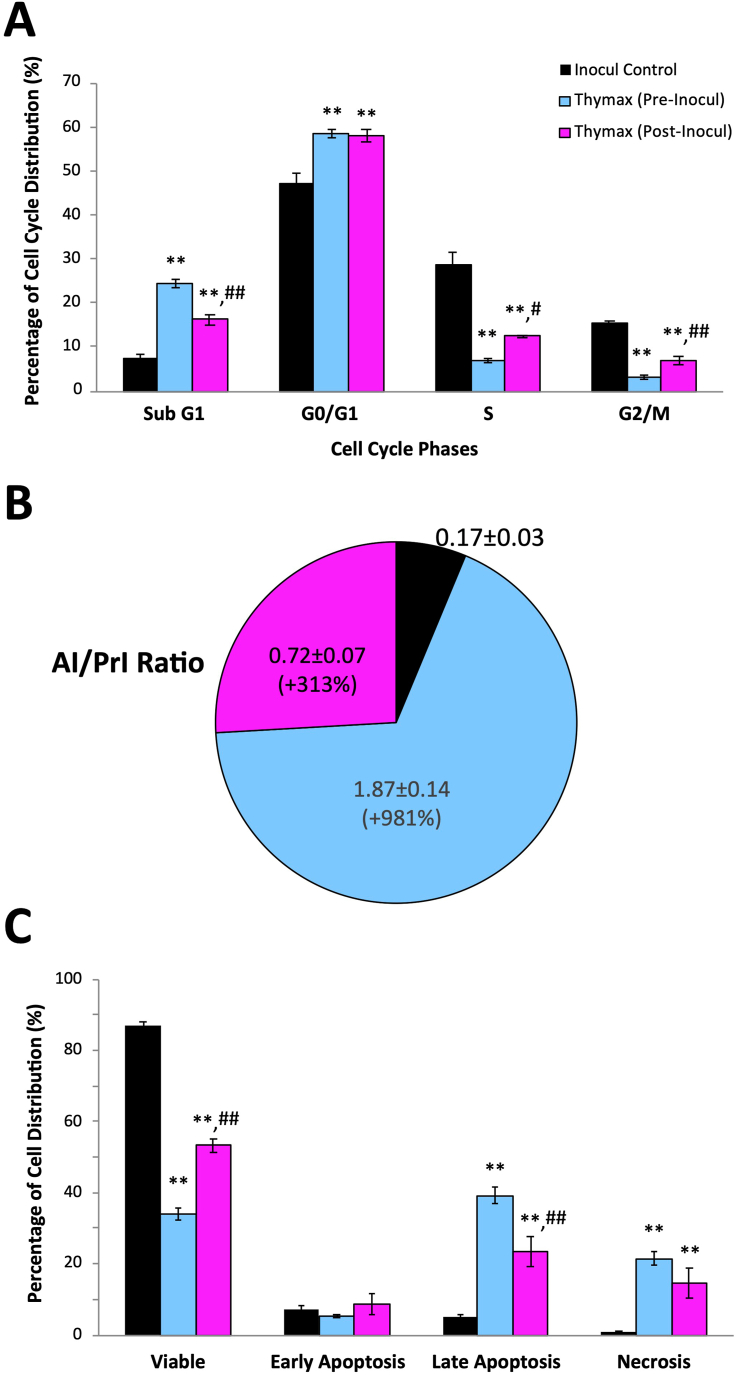
(A) Effect of Thymax on cell cycle progression and apoptosis in tumor tissues of the different groups as detected by flow cytometry. Each value represents the mean ± SE of 10 mice in each group. (B) Effect of Thymax on AI/PrI ratio in tumor tissues of the different groups. Each value represents the percentage change of the mean of 10 mice/group versus the Inocul Control group. (C) Effect of Thymax on apoptosis as detected by Annexin V/PI. Each value represents the mean ± SE of 6 mice. ∗∗ Significantly different from the Inocul Control group at *p < 0.01* level. ^##,#^ Significantly different from the Thymax (Pre-Inocul) group at *p < 0.01,0.05* level, respectively.

Mice under different treatment conditions were examined for cell cycle progression using flow cytometry of PI-stained cells (Figure S1 shows a representative plot). Data in Figure 2A show a significant increase in the hypodiploid cell percentages in the sub-G1 phase for the Thymax Pre-Inocul group (3.3 fold) and Post-Inocul group (2.2 fold) (p < 0.01) relative to the Inocul Control group. Pre- and post-treatment with Thymax also arrested the cell cycle in the G0/G1 phase, demonstrating a significantly (p < 0.01) increased cell population in the G1 phase (24.2% and 23.2%, respectively), versus the Inocul Control group, followed by reduced numbers in the S and G2/M phases versus Inocul Control animals.

### 3.3 Apoptosis index/proliferation index ratio (AI/PrI)

Data in Figure 2B show that Thymax treatment changed the AI/PrI ratio. Thymax treatment increased AI/PrI by 11 fold and 4 fold (p < 0.01) for Pre-Inocul and Post-Inocul groups, respectively, in comparison with the Inocul Control group.

### 3.4 Quantitative determination of apoptosis by AnnexinV/PI staining

AnnexinV/PI double staining was performed to quantitatively analyze apoptosis via flow cytometry (Figure S2 shows a representative plot). Figure 2C reveals that Thymax pre- and posttreatment decreased the percentage of viable cells by 61% and 39% (p < 0.01), respectively, relative to the Inocul Control group. Pre- or posttreatment with Thymax showed no significant difference for the early apoptosis detection relative to the Inocul Control group, but pre- and posttreatment with Thymax increased the late apoptotic population by 8 fold and 4.8 fold (p < 0.01), respectively, relative to the Inocul Control group. The overall total apoptotic population of Thymax pre- and posttreatment groups (the sum of early and late apoptosis) was 263% and 162% (p < 0.01), respectively, of the Inocul Control group (data not shown), and the necrotic population of Thymax Pre- and Post-Inocul groups revealed an increase of 27.6 fold and 18.9 fold (p < 0.01), respectively, as compared to the Inocul Control group.

### 3.5 The expression of cell cycle and apoptotic regulators by flow cytometric and western blotting analysis

**Figure 3:**
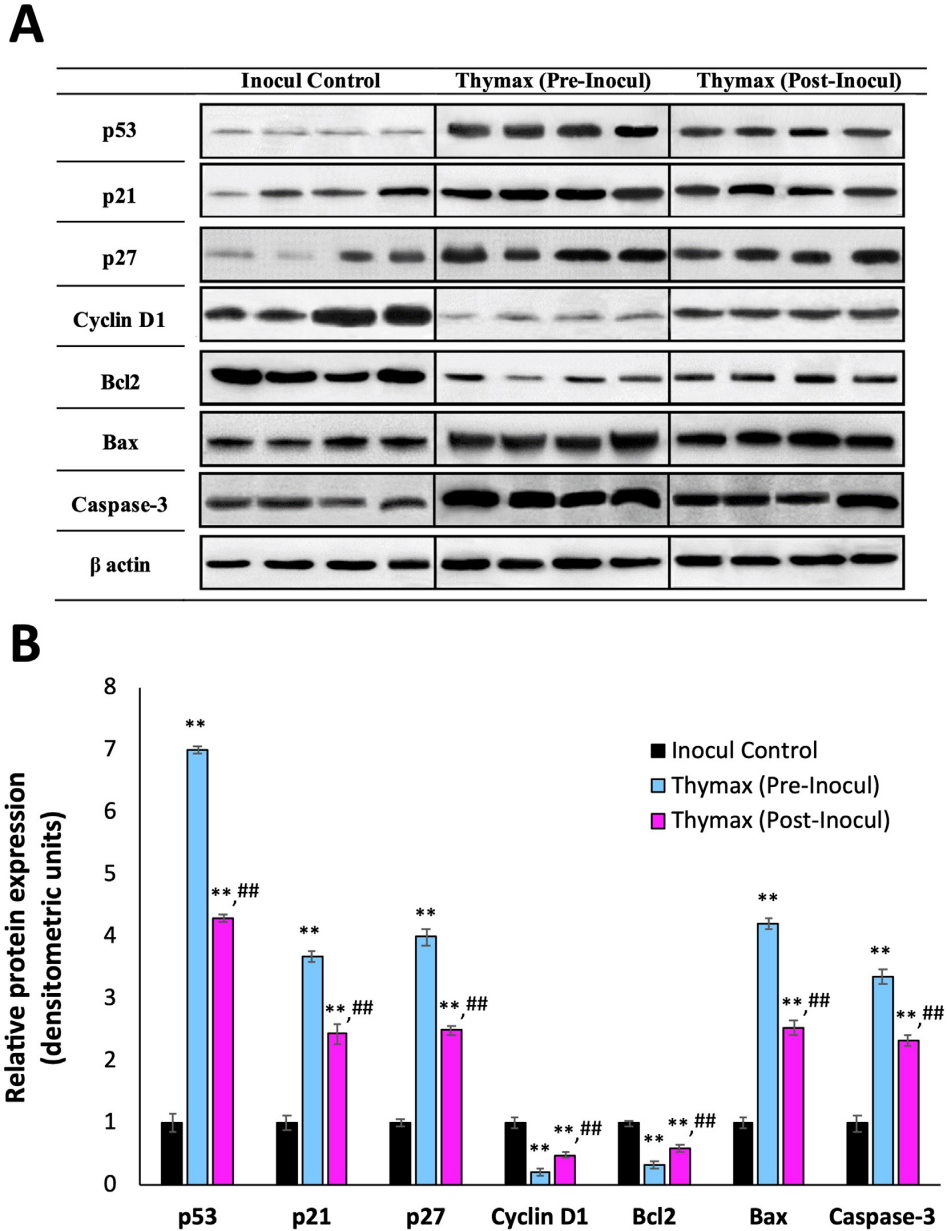
(A) Effect of Thymax on the expression level of cell cycle and apoptotic regulators in tumor tissues of EAC-bearing mice as determined by Western blot analysis using β-actin as loading control. Lanes 1–4: Inocul Control group; lanes 5–8: Thymax (Pre-Inocul) group; lanes 9–12: Thymax (Post-Inocul) group. Band intensity reveals relative changes in protein expression using the Inocul Control group as a control. (B) Graphical representation: Data represents the band relative densities expressed as mean ± SE of 4 mice in each group. ∗∗ Significantly different from the Inocul Control group at *p < 0.01* level. ^##^ Significantly different from the Thymax (Pre-Inocul) group at *p < 0.01* level.

Flow cytometric analysis (Table 1, with representative plots in Figures S3–S9) revealed that the protein level percentages of p27, p21, and p53 in the tumor cells of mice post-treated with Thymax were upregulated relative to the tumor tissues of Inocul Control to record an increase of 2.4 fold, 4.4 fold, 2.4 fold, and 4.4 fold, respectively. However, pretreatment with Thymax showed a more noticeable upregulation, with the levels increasing by 3.4 fold, 3.6 fold, and 6.8 fold, respectively, versus the Inocul Control group. In the same time, Cyclin D1 expression was downregulated in tumor cells of Thymax post-treated and pretreated groups (p < 0.01) by 45.7% and 75.7%, respectively, versus Inocul Control mice. Thymax posttreatment showed marked upregulation (p < 0.01) for Bax and caspase-3 protein expression by 2.5 fold and 2.4 fold, respectively, and marked downregulation in Bcl-2 protein expression by 41.9% (p < 0.01) versus Inocul Control. Thymax pretreatment revealed more marked upregulation in tumor cells for Bax expression by 4.3 fold and caspase-3 by 3.4 fold, while the expression of Bcl-2 was downregulated by 67.9% versus Inocul Control mice. Furthermore, pretreatment and posttreatment with Thymax revealed a high increase in Bax/Bc12 ratio versus the Inocul Control group. Western blotting and densitometric analysis confirmed these results; the protein expression levels presented a trend similar to flow cytometric analysis, as shown in Figure 3.

**Table 1:** Effect of Thymax on the expression levels of cell cycle and apoptotic regulators as determined by flow cytometry.

Parameter	Inocul Control	Thymax (Pre-Inocul)	Thymax (Post-Inocul)
p53	9.28 ± 0.52	63.40^a^ ±1.79	40.45^a^^,^^b^ ±1.43
% change from Inocul Control	-	+583.19%	+335.88%
p21	15.83 ± 0.82	57.33^a^ ±0.64	37.88^a^^,^^b^ ±0.8
% change from Inocul Control	-	+262.16%	+139.29%
p27	16.78 ± 0.65	66.33^a^ ±1.55	40.43^a^^,^^b^ ±0.42
% change from Inocul Control	-	+295.29%	+140.94%
Cyclin D1	78.4 ± 1.71	19.06^a^ ±1.01	42.58^a^^,^^b^ ±1.32
% change from Inocul Control	-	-75.69%	-45.69%
Bax	13.93 ± 2.1	59.51^a^ ±2.52	35^a^^,^^b^ ±1.21
% change from Inocul control	-	+327.21%	+151.26%
Bcl-2	76.33 ± 1.57	24.53^a^ ±0.82	44.35^a^^,^^b^ ±1.05
% change from Inocul Control	-	-67.86%	-41.9%
Bax/Bcl-2	0.183 ± 0.02	2.45^a^ ±0.17	0.79^a^^,^^b^ ±0.03
% change from Inocul Control	-	+1238.89%	+331.69%
Caspase-3	17.11 ± 2.11	58.11^a^±3.19	41.06^a^^,^^b^ ±1.84
% change from Inocul Control	-	+239.63%	+139.98%

Each value represents the mean ± SE of 6 mice in each group**.**

a Significantly different from Inocul Control group at *p < 0.01* level.

b Significantly different from Pre-Inocul group at *p < 0.01* level.

### 3.6 Detection of cell proliferation markers Ki-67 and PCNA

**Figure 4:**
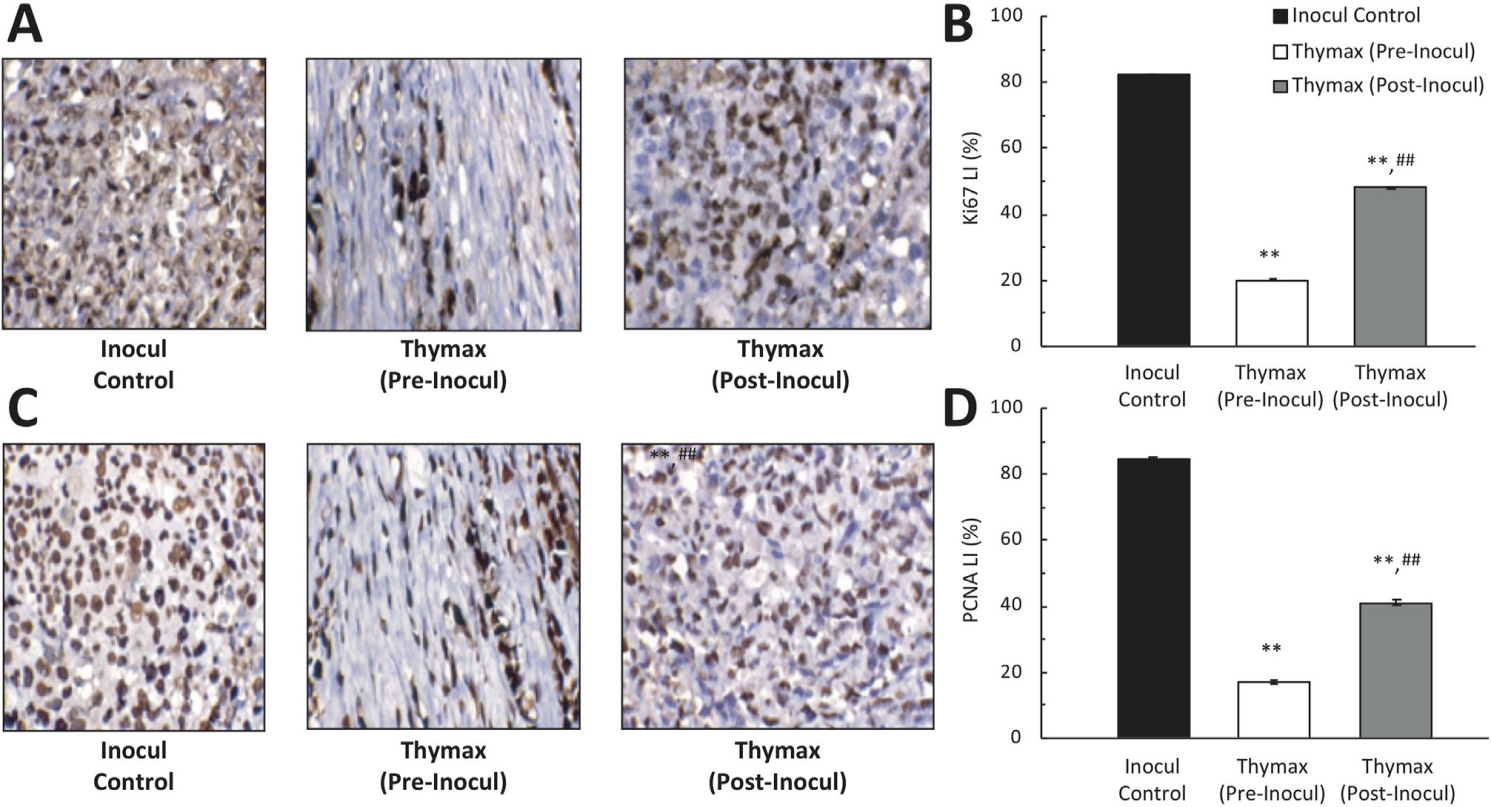
(A) Immunohistochemical detection of Ki-67 protein expression in tumor tissues, x400. (B) Corresponding Ki-67 labeling index values. (C) Immunohistochemical examination of PCNA expression in tumor tissues, x400. (D) Corresponding PCNA labeling index values. Each value represents the mean ± SE of 6 mice in each group. ∗∗ Significantly different from the Inocul Control group at *p < 0.01* level. ^##^ Significantly different from the Thymax (Pre-Inocul) group at *p < 0.01* level.

The effect of Thymax on Ki-67 and PCNA protein expression in tumor tissues of the Inocul Control mice was examined with flow cytometry measurements of protein expression in tumor tissues, immunohistochemical analysis, and measurements of labeling indices. Table 2 (and representative plots shown in Figures S10–S11) show that Thymax treatment significantly decreased Ki-67 expression by 70.0% for the Thymax Pre-Inocul group and 46.0% for the Thymax Post-Inocul group relative to the Inocul Control group. In addition, pre- and posttreatment with Thymax demonstrated a significant decrease of 80.2% and 54.4% in levels of PCNA expression relative to the Inocul Control group. Furthermore, tumor tissues of untreated Inocul Control showed strong Ki-67 and PCNA protein expression within the nuclei of the neoplastic cells. Thymax pretreatment showed marked decrease in both markers’ labeling index by 75.8% and 79.9% for Ki-67 and PCNA, respectively, as compared with the Inocul Control. Thymax posttreatment also showed a noticeable decrease in the immunoreactivity by 41.5% and 51.5% for the labeling index of Ki-67 and PCNA, respectively, as compared with the Inocul Control group (Figure 4).

**Table 2:** Effect of Thymax on Ki-67 and PCNA protein expression in tumor tissues as determined by flow cytometry.

Parameter	Inocul Control	Thymax (Pre-Inocul)	Thymax (Post-Inocul)
Ki-67 expression	77.65 ± 3.38	23.31^a^ ± 0.8	41.93^a^^,^^b^ ± 0.78
% change from Inocul Control	-	-69.98%	-46.00%
PCNA	85.71 ± 0.89	17.01^a^ ± 1.24	39.06^a^^,^^b^ ± 1.32
% change from Inocul Control	-	-80.15%	-54.43%

Each value represents the mean ± SE of 6 mice in each group**.**

a Significantly different from Inocul Control group at *p < 0.01* level.

b Significantly different from Pre-Inocul group at *p < 0.01* level.

### 3.7 Detection of DNA damage by gel-electrophoresis

**Figure 5:**
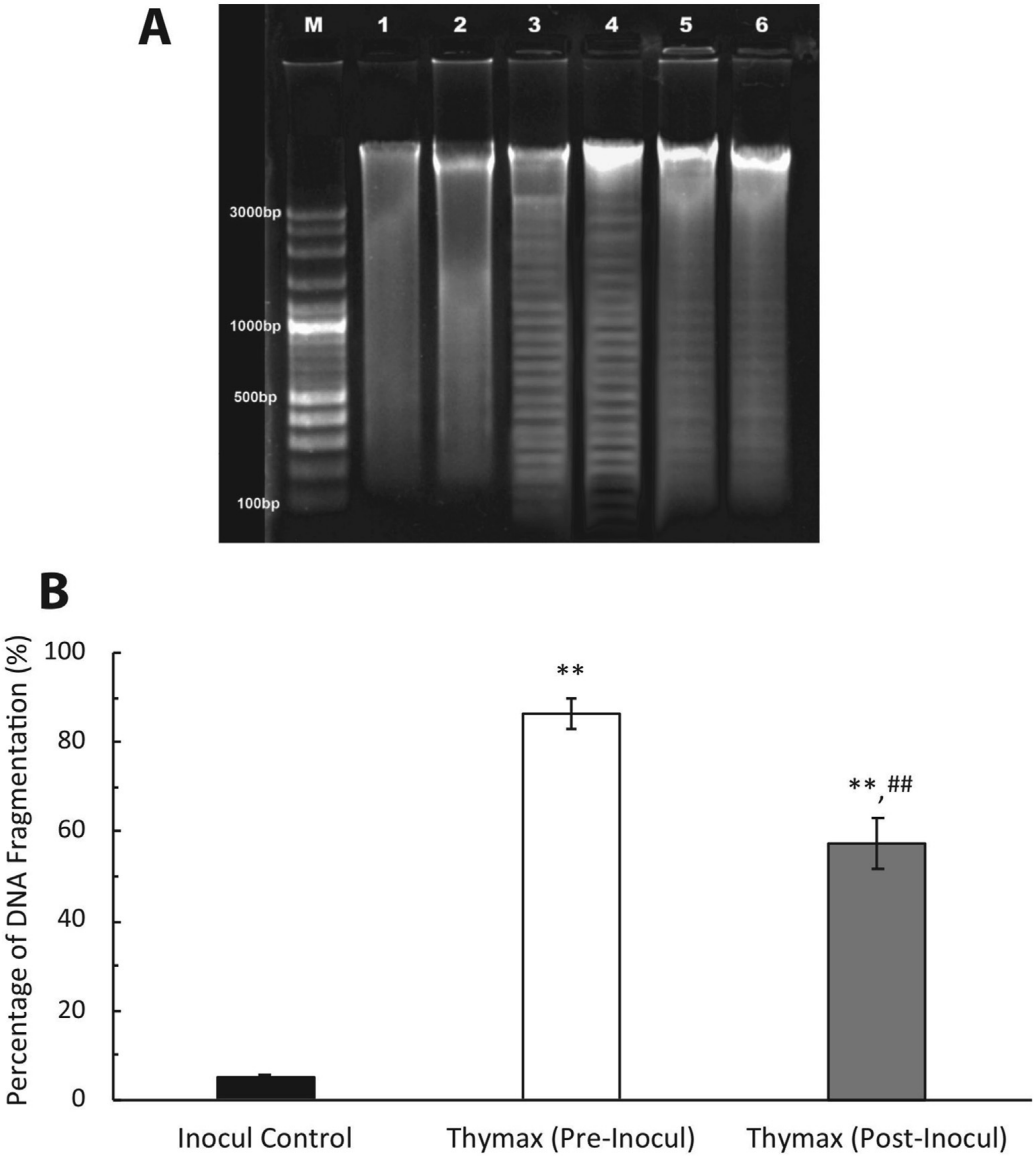
Effect of Thymax on DNA damage. (A) Gel electrophoresis analysis of genomic DNA fragmentation in tumor tissues of mice treated with Thymax, with different lanes profiling the genomic DNA on agarose gel. Lane M: 100bp molecular weight DNA ladder marker; Lane 1&2: Inocul Control group; Lane 3&4: Thymax (Pre-Inocul) group; Lane 5&6: Thymax (Post-Inocul) group. (B) Percentage of DNA fragmentation obtained by gel electrophoresis in tumor tissues of mice treated with Thymax and analyzed by ImageJ software. Each value represents the mean ± SE of 3 gel electrophoresis runs. ∗∗ Significantly different from the Inocul Control group at *p < 0.01* level. ^##^ Significantly different from the Thymax (Pre-Inocul) group at *p < 0.01* level.

DNA agarose electrophoresis was used to measure nuclear DNA fragmentation, with DNA laddering giving the characteristic signification of apoptosis. As shown in Figure 5, the DNA fragmentation in Inocul Control tumor cells produced a percent fragmentation of 5.99 ± 1.97 and exhibited no ladder formation. Conversely, broken DNA strands in tumor cells of the Thymax Post-Inocul group recorded a percent fragmentation of 59.9 ± 2.6, a 10-fold increase over the Inocul Control group. The Thymax Pre-Inocul group showed even more DNA damage and the highest DNA laddering percentage (90.4 ± 2.8), representing a 15-fold increase over the Inocul Control group.

## 4 Discussion

Results of this study showed that Thymax supplementation induces chemoprotective effects against EAC bearing mice. Pretreatment of mice with Thymax markedly delayed tumor growth and reduced tumor incidence by 38.9% as recorded on day 30. Significant inhibition in percent tumor volume reached 55.0% and 90.5% for post-inoculation and pre-inoculation, respectively. The anticancer effect of Thymax is in correspondence with previous in vitro and in vivo studies demonstrating the therapeutic effects of Thymax [24, 25, 26], where Thymax induced apoptosis in vitro in human breast cancer cells by a mitochondrial pathway [24] and activated human monocyte-derived DCs [26], and in vitro was shown to have the ability to correct age-associated functional decline of murine immune cells in mice [25]. Our findings also agree well with other studies on different thymus products such as Thymosin alpha1 (Tα1) which demonstrated significant suppression in the tumor growth of animals bearing lung and breast cancer [13], as well as with other studies treating patients with different types of malignancies including hepatocellular carcinoma [37], non–small-cell lung cancer [14], and melanoma [17]. Furthermore, immunotherapy with thymic humoral factor-gamma 2 (THF-γ2) has been used for cancer patients as an chemotherapy adjunct [38, 39, 40, 41]. In addition, combining chemotherapy with thymic hormones has been shown to significantly increase survival in mice bearing tumor [42]. The mechanisms by which Thymax induces tumor regression in EAC bearing mice are unclear, but they may involve cell cycle arrest and apoptotic properties of this agent against cancer cells.

Cell cycle progression was analyzed and the viable cell count was measured to be significantly inhibited. Furthermore, the cell cycle distribution data show that Thymax treatment results in a dramatically elevated cell number in the G0/G1 phase, along with a corresponding cell number decrease in other phases. The appearance of a significant sub-G0/G1 peak indicated that there was an accompanying induction of apoptosis, and this was confirmed by AnnexinV/PI assay and a DNA laddering pattern detected by agarose gel electrophoresis. Endogenous nucleases that are activated during apoptosis cause DNA degradation that is signaled by this hypodiploidy [43]. In addition, treatment with Thymax induced a marked increase in the apoptosis index/proliferation index (AI/PrI) ratio by 4.1 and 10.8 fold for post- and pre-treatment, respectively, versus control.

In this study, we examined several key regulatory molecules that control the apoptotic pathways, including p53, anti-apoptotic Bcl-2, proapoptotic BAX, and caspase-3. Furthermore, we examined the ability of Thymax to target these regulatory molecules for apoptosis induction. Treatment with Thymax decreased the mitochondrial membrane potential (MMP) (data not shown), activated caspase-3, and increased the Bax/Bcl-2 ratio in neoplastic cells, indicating that Thymax induces apoptosis via an intrinsic pathway. Groc et al. [44] have reported an increased Bax/Bcl-2 ratio during apoptotic death, and an increased ratio has also been found in accordance with an apoptotic effect by Tα1 in human leukemia cell lines via up-regulation of Fas/Apol (CD95) and decreased Bcl-2 expression [45]. With regard also to caspases, earlier in vitro studies have also shown that caspases 8 and 9 activation occurs in human MCF-7 breast cancer cells [24], suggesting that for apoptosis induced by Thymax, pathways that are both dependent on and independent of caspases may play a role.

Targeting the cell cycle is a new approach to cancer therapy. Specific cyclins and cyclin-dependent kinases (CDKs) act together in a highly orchestrated set of steps called the cell cycle to carry out cell division [46]. Results of the present study showed that Thymax treatment significantly decreased tumor cell proliferation as indicated by marked suppression in the expression of PCNA, Ki-67, and Cyclin D1 in cancer cells. PCNA and Ki-67 are molecular markers that are useful tools for studying cell proliferation, with their possible apparent expression in neoplasm indicating cell division and proliferation, and Cyclin D1is a protein that promotes proliferation and is mostly expressed during the G1-S phase transition [47, 48]. Our results also showed concomitant upregulation in the expression of p53, p21, and p27 relative to untreated control. Since p21 inhibits the CDKs that regulate the cell cycle [49], up-regulation of p21 blocks the progression of the cell cycle and thereby inhibits cells. The progression of the cell cycle towards the S phase and the initiation of mitosis depends on the activation of cyclins that bind to CDKs. Since human cancer is often caused by uncontrolled CDK activity, cell-cycle inhibitors such as the p21 and p27 Cip/Kip proteins strictly regulate CDK function. p21 and p27 can arrest the cell-cycle by binding to cyclin–CDK complexes, thereby inhibiting their catalytic activity [50].

In the present study, there was a significant decrease in the body weight of the Inocul Control group in comparison with other groups. However, Thymax treatment maintained normal body weight gain comparable with the normal control values (data not shown). Decreased appetite and food intake may contribute to the weight loss, which could be attributed to the formation of tumors [51]. Feeding, drinking, and patterns of life activity were observed to be normal for all animals over the course of the 30-day treatment period. This data further confirm earlier findings showing no abnormal organ pathology at one month post Thymax supplementation [25], indicating that Thymax could potentially be a non-toxic, nonthreatening, and safe agent for humans. We also hope to conduct thorough investigations in the near future with regard to the fraction of thymus crude extract that is optimal for treatment.

## 5 Conclusion

We have demonstrated that Thymax reduces tumor incidence and growth in EAC cells by suppressing cancer cell proliferation and inducing apoptosis through a mitochondrial-dependent pathway. Data in the current study and the lack of any appearance of side effects suggest that much interest should be given to this novel Thymax treatment, and its effect on other cancer models should be investigated. The results of the current study indicate Thymax's chemopreventive potential and suggest that Thymax may be applicable for cancer prevention and/or treatment in clinical trials.

## Declarations

### Author contribution statement

Nariman K. Badr El-Din and Azza I. Othman: Conceived and designed the experiments; Performed the experiments; Analyzed and interpreted the data.

Maggie E. Amer: Performed the experiments; Analyzed and interpreted the data.

Mamdooh Ghoneum: Conceived and designed the experiments; Analyzed and interpreted the data.

### Funding statement

This research did not receive any specific grant from funding agencies in the public, commercial, or not-for-profit sectors.

### Data availability statement

Data will be made available on request.

### Declaration of interests statement

The authors declare no conflict of interest.

### Additional information

No additional information is available for this paper.

## Figures and Tables

**S1-Cell-Cycle:**
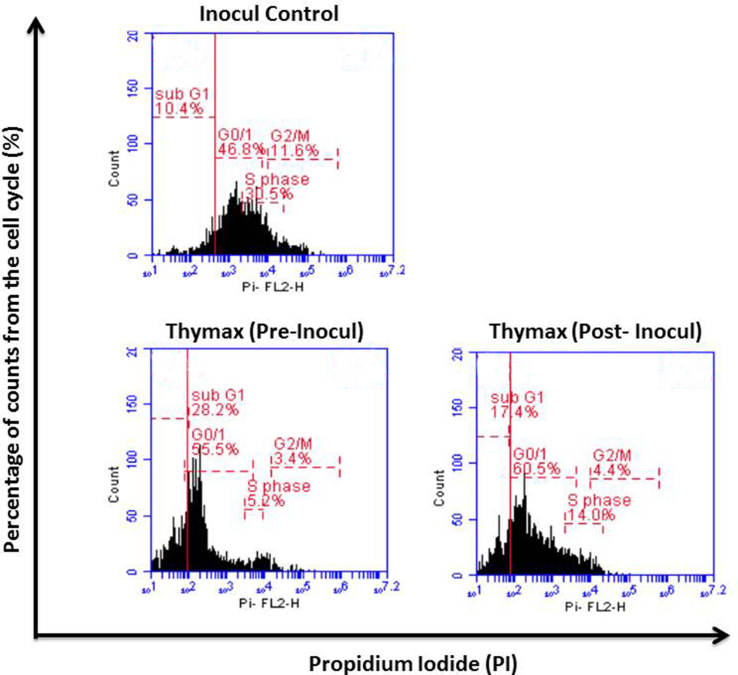
Supplementary Figure 1. Representative flow cytometry result for cell cycle.

**S2-Annexin-V:**
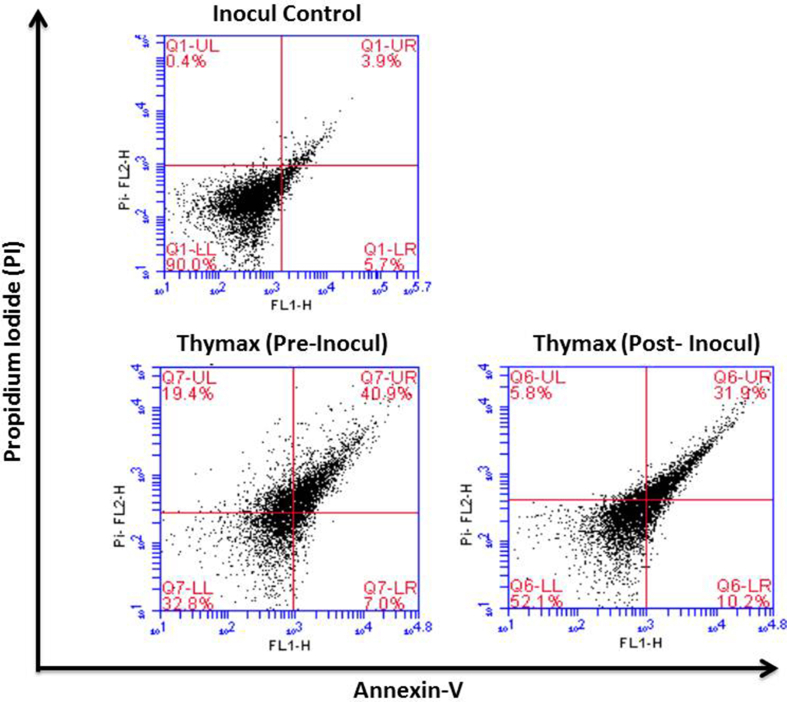
Supplementary Figure 2. Representative flow cytometry result for Annexin V/PI.

**S3-Bax-:**
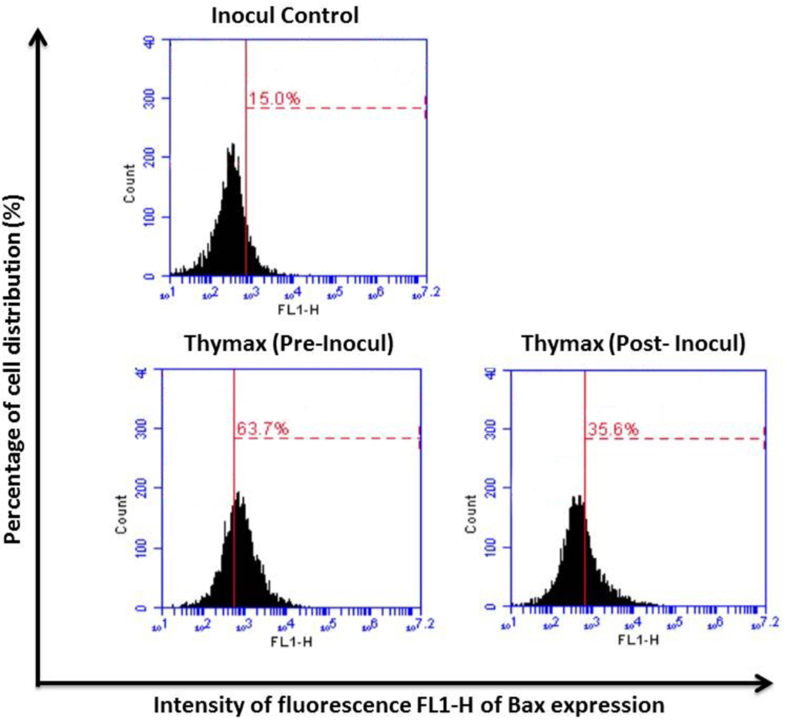
Supplementary Figure 3. Representative flow cytometry result for Bax.

**S4-Bcl-2-:**
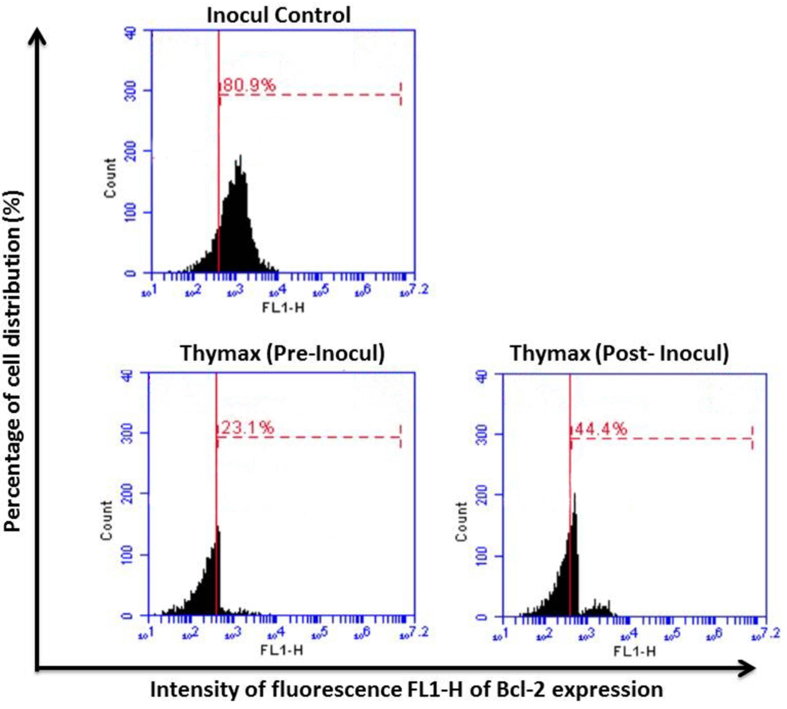
Supplementary Figure 4. Representative flow cytometry result for Bcl-2.

**S5-Caspase-3:**
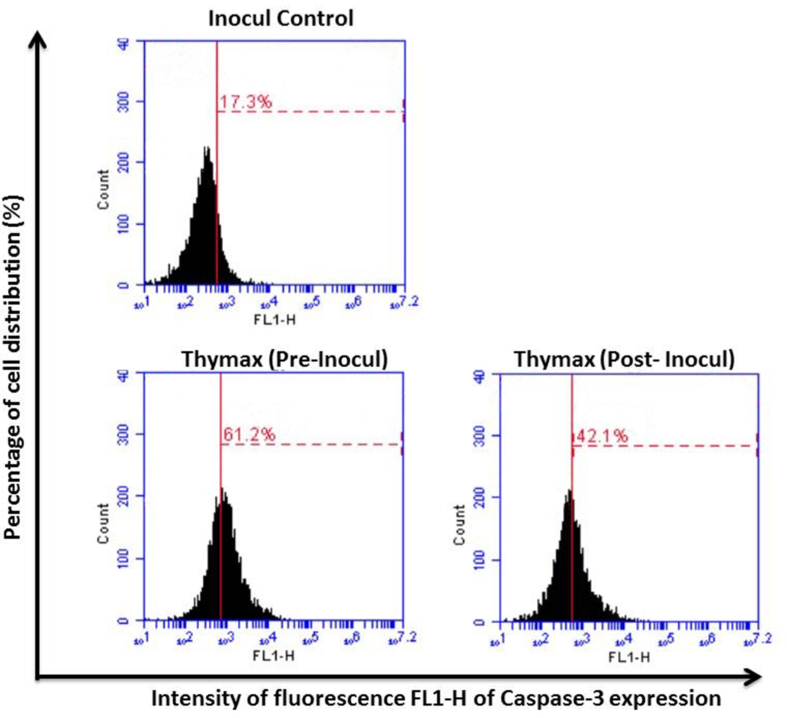
Supplementary Figure 5. Representative flow cytometry result for Caspase-3.

**S6-Cyclin D1:**
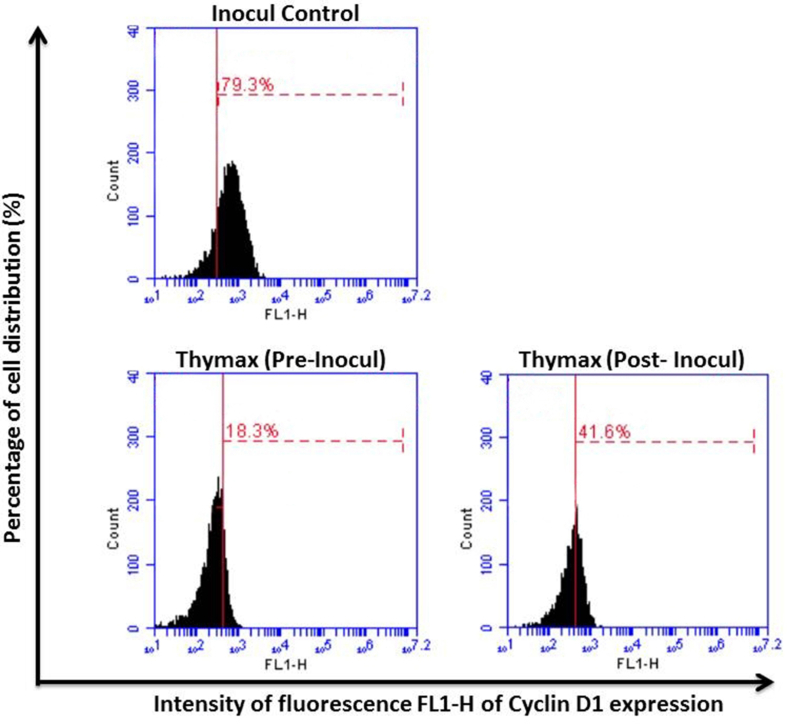
Supplementary Figure 6. Representative flow cytometry result for Cyclin D1.

**S7-p21:**
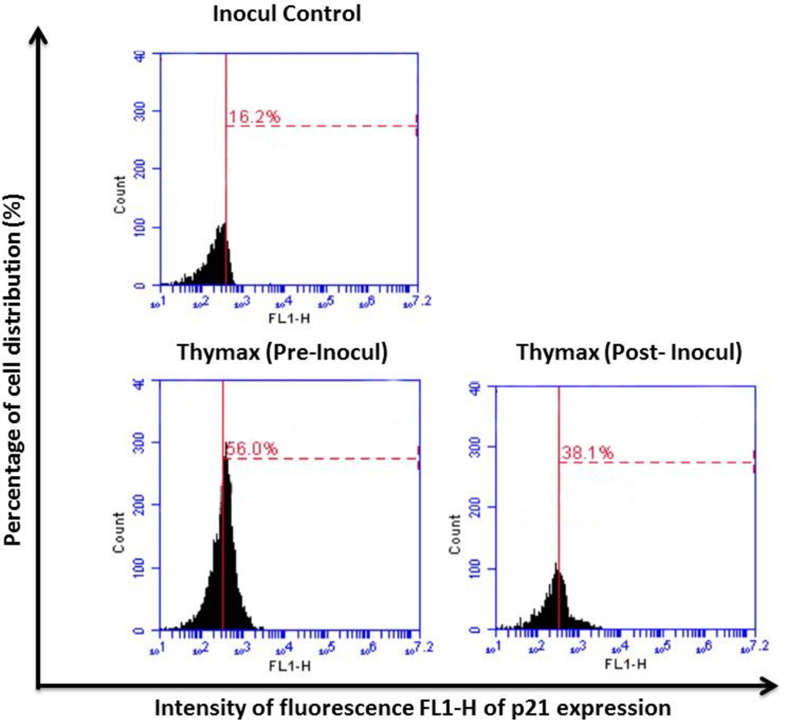
Supplementary Figure 7. Representative flow cytometry result for p21.

**S8-p27:**
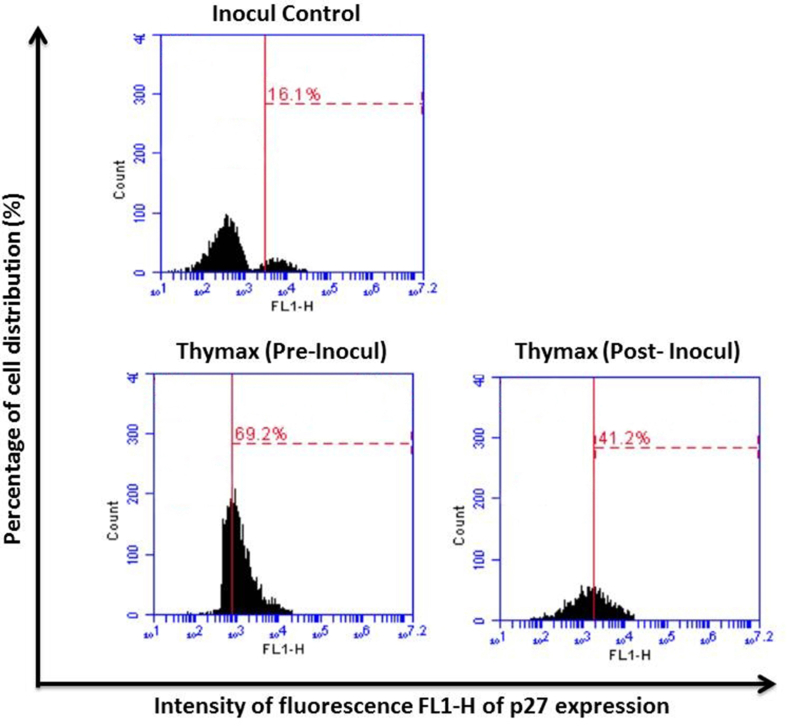
Supplementary Figure 8. Representative flow cytometry result for p27.

**S9-p53:**
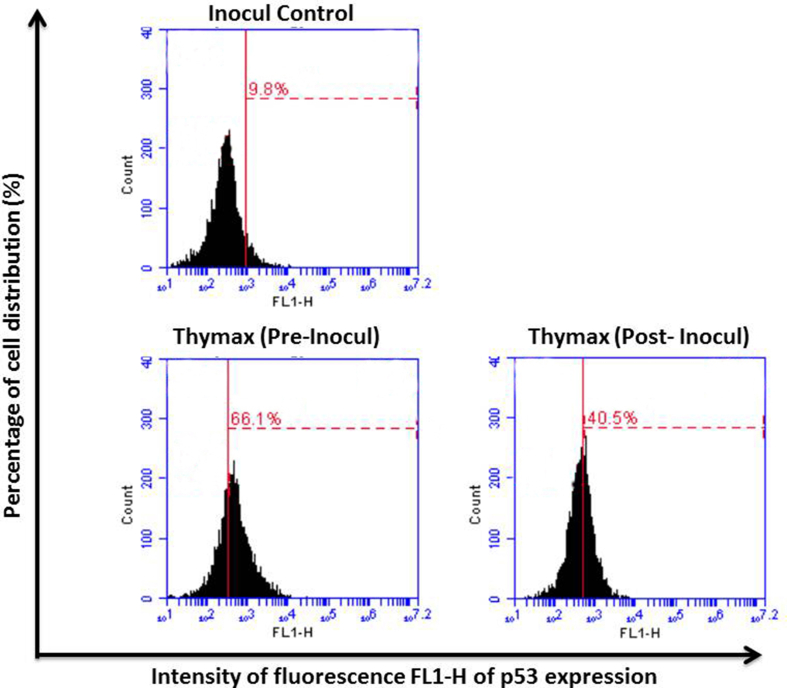
Supplementary Figure 9. Representative flow cytometry result for p53.

**S10-KI-67:**
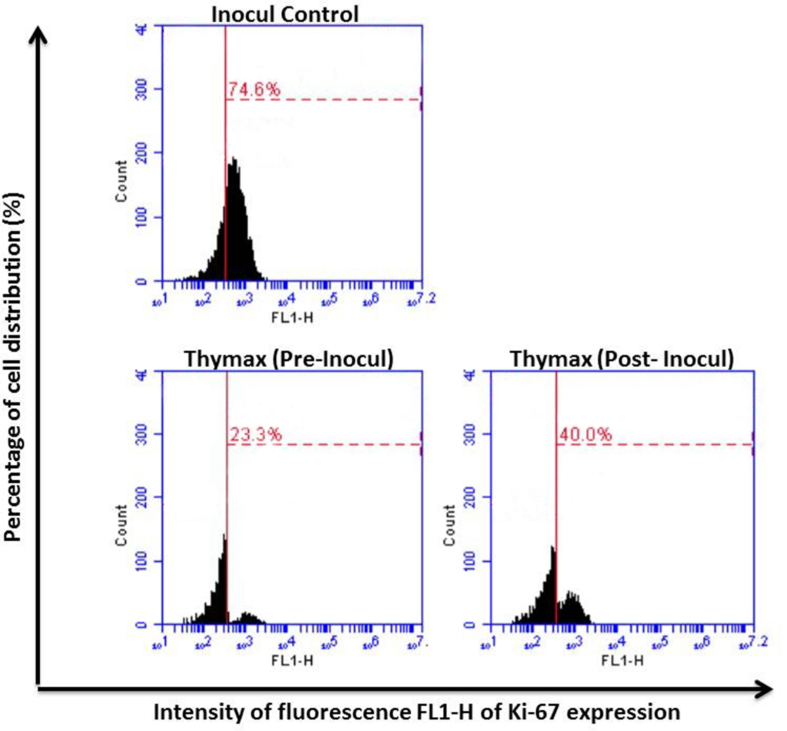
Supplementary Figure 10. Representative flow cytometry result for Ki-67.

**S11-PCNA:**
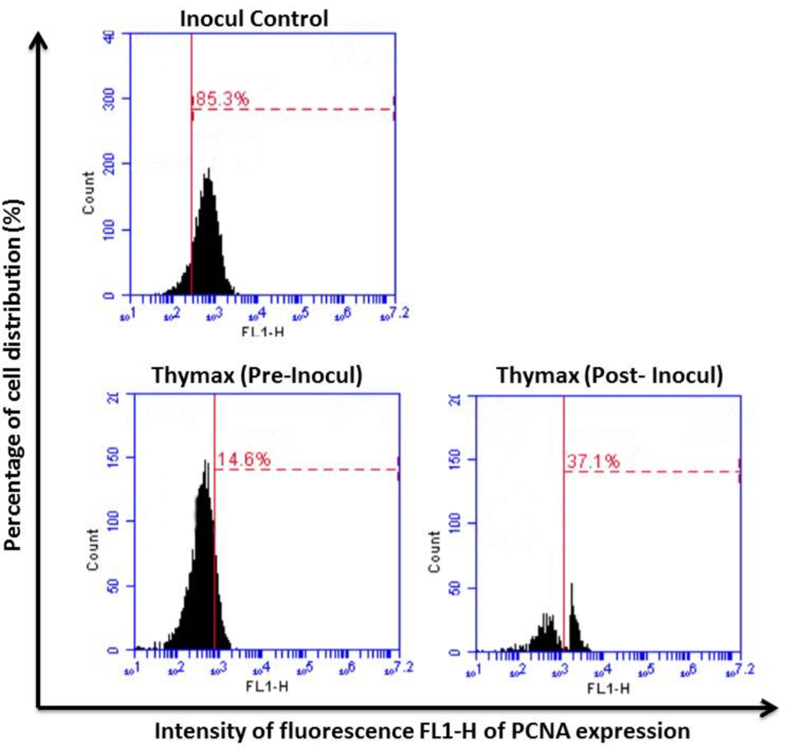
Supplementary Figure 11. Representative flow cytometry result for PCNA.

## Appendix A Supplementary data

SupplementaryFile1-011222 SupplementaryFile1-011222
