# Supplementary material for: Thymax, a gross thymic extract, exerts cell cycle arrest and apoptosis in Ehrlich ascites carcinoma in vivo
Source: Heliyon. 2022 Mar 5;8(3):e09047. doi: 10.1016/j.heliyon.2022.e09047 (PMC8920936; doi:10.1016/j.heliyon.2022.e09047)
Supplement: SupplementaryFile1-011222 [file mmc1.docx]

**Preliminary study**

**Purpose**: In our earlier study (24), we showed that Thymax induced phenotypic correction of age-associated functional decline in immune cells in mice by using a daily oral dose to mice of 0.33 ml/mouse for four weeks. For the current study we conducted a preliminary experiment to determine the optimum dose in which to test the anticancer effect of Thymax. **Materials & Methods**: Thirty-two Swiss albino mice weighing ~22 g were inoculated intramuscularly in the thigh with 2.5 x 10^6^ viable EAC cells/animal. On day 9 after tumor cell inoculation, mice bearing palpable tumors were divided into 4 groups (8 mice/group). We used three different doses of Thymax, with the lowest dose being representative of the dose used in Ghoneum et al. (24): 0.3, 0.6, and 0.9 ml/day given orally until day 27 after tumor cells inoculation. Tumor volume was monitored at different time intervals and compared with the control untreated group. **Results:** Treatment with Thymax markedly reduced tumor volume by 55.8% and 63.03% (p<0.01) for doses of 0.6 and 0.9 ml/day/mouse, respectively, relative to the control untreated mice (see Figure). There was no significant difference between these two doses. In the current study, we preferred to use the medium dose of Thymax (0.6 ml/mouse/day). The Thymax solution was prepared by dissolving 10 mg in 50 ml water. For a mean mouse body weight of approximately 22 g and a dose of 0.6 ml/mouse = 0.12 mg/mouse, the matching dose can then be calculated to be the present dose of 5.45 mg/kg BW.

**
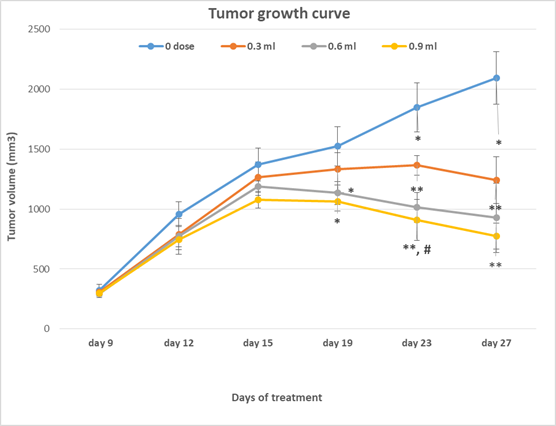
**

Figure: Mice in the preliminary study were inoculated intramuscularly with Ehrlich ascites carcinoma cells to induce solid tumor. Palpable tumors were treated daily from day 9 to day 27 after tumor cell inoculation with 0.3, 0.6, and 0.9 ml/day/mouse doses of Thymax and compared to the untreated control group (no Thymax). Data are expressed as mean ± SEM. *, ** Significantly different from control at p<0.05 and p<0.01 level, respectively. ^#^ Significantly different from the 0.6 ml/day/mouse dose at p<0.05 level at corresponding time points.
